# Infrahepatic inferior vena cava clamping with Pringle maneuvers for laparoscopic extracapsular enucleation of giant liver hemangiomas

**DOI:** 10.1007/s00464-016-5396-6

**Published:** 2017-01-27

**Authors:** Wanguang Zhang, Jian Wang, Changhai Li, Zhanguo Zhang, Najib Isse Dirie, Hanhua Dong, Shuai Xiang, Wei Zhang, Zhiwei Zhang, Bixiang Zhang, Xiaoping Chen

**Affiliations:** 1 Department of Surgery, Hepatic Surgery Center, Tongji Hospital, Tongji Medical College, Huazhong University of Science and Technology, Wuhan, China; 2 Department of Surgery, Tongji Hospital, Tongji Medical College, Huazhong University of Science and Technology, Wuhan, China

**Keywords:** Giant liver hemangiomas, Extracapsular enucleations, Laparoscopic hepatectomy, Inferior vena cava clamping, The Pringle maneuvers, Bleeding control

## Abstract

**Background:**

This study aimed to determine the feasibility of the extracapsular enucleation method for giant liver hemangiomas by infrahepatic inferior vena cava (IVC) clamping and the Pringle maneuver to control intraoperative bleeding under laparoscopic hepatectomy.

**Methods:**

From January 2012 to January 2016, 36 patients underwent laparoscopic extracapsular enucleation of giant liver hemangiomas. Patients were divided into two groups: infrahepatic IVC clamping + Pringle maneuvers group (IVCP group, *n* = 15) and the Pringle maneuvers group (Pringle group, *n* = 21). Operative parameters, postoperative laboratory tests, and morbidity and mortality were analyzed.

**Results:**

The mean size of liver hemangiomas was 13.3 cm (range 10–25 cm). Infrahepatic IVC clamping + the Pringle maneuvers with laparoscopic extracapsular enucleation significantly reduced intraoperative blood loss (586.7 vs 315.3 mL, *p* < 0.001) and transfusion rates (23.8 vs 6.7%, *p* = 0.001), compared with the Pringle maneuver alone. The gallbladder was retained in both groups. The mean arterial pressure (MAP) in Pringle group remained virtually stable before and after clamping of hepatic portal, while it was significantly decreased after IVC clamping in IVCP group than that pre-clamping (*p* < 0.001). The heart rate of all patients was significantly increased after clamping when compared to pre-clamping heart rates (*p* < 0.001). Once vascular occlusion was released, MAP returned to normal levels within a few minutes. There were no significant differences in postoperative complications between two groups. The vascular occlusion techniques in both groups had no serious effect on postoperative of hepatic and renal function.

**Conclusions:**

Extracapsular enucleation with infrahepatic IVC clamping + the Pringle maneuver is a safe and effective surgical treatment to control bleeding for giant liver hemangiomas in laparoscopic hepatectomy.

Cavernous hemangioma is the most common benign hepatic tumor, with an estimated prevalence of 3–20% [1]. For most patients, the natural history of cavernous hemangiomas in the liver remains constant in size over time and a giant hemangioma is no longer a necessary indication for surgery based on its size alone [2–6]. Routine follow-up observations and corresponding imaging examinations of asymptomatic lesions are generally adequate. However, some cavernous hemangiomas may gradually grow into a large size and subsequently become symptomatic [7]. The presence of progressive abdominal symptoms, spontaneous or traumatic rupture, rapidly enlarging lesions, Kasabach-Merritt syndrome, and uncertain diagnosis of liver hemangiomas are indications for surgical resection [8].

Surgery remains the solely curative treatment for giant liver hemangiomas. Anatomical liver resection and extracapsular enucleation are the most commonly used surgical approaches for giant hemangiomas [6]. Anatomical resection involves resection of the hemangioma along with the ligation of related hepatic pedicle and division of hepatic parenchyma. This method results in loss of normal liver tissues and gallbladder, especially right hemihepatectomy. Hence, extracapsular enucleation of hemangioma is the preferable treatment option. But for a benign tumor, the distress caused by the open surgery may be far greater than tumor-related discomfort. Most surgeons advocated laparoscopic liver resection of liver hemangiomas because of faster postoperative recovery, fewer overall complications, and shorter hospital stay over open surgery [9]. However, control of bleeding in laparoscopic hepatectomy is much more technically demanding than that in open [10]. There are many ways to control surgical bleeding, such as intermittent inflow occlusion of the portal triad (Pringle maneuver), and reduction of central venous pressure (CVP), which have been proved to reduce blood loss during laparoscopic hepatectomy [10–12]. As is known to all, to safely perform complicated hepatectomy in open surgery, clamping of the infrahepatic inferior vena cava (IVC) is an alternative technique to reduce CVP and blood loss during resection of the liver parenchyma [13–15]. Laparoscopic hepatic hemangioma enucleation is still more difficult to control bleeding, compared with open surgery. Whether infrahepatic IVC clamping could be applied into laparoscopic hepatectomy remains unknown.

The present study aimed to evaluate safety and efficacy of selective infrahepatic IVC clamping method to control bleeding in laparoscopic extracapsular enucleation for giant liver hemangiomas.

## Methods

From January 2012 to January 2016, 36 patients with giant liver hemangiomas underwent laparoscopic liver resection at Tongji Hospital of Huazhong University of Science and Technology, Wuhan, China. All of the procedures were performed by the same surgeon. This study was approved by the Ethics Committee for Clinical Pharmacology in Tongji Medical College. Giant liver hemangiomas were defined by a diameter larger than 10 cm. Based on the maneuvers of hepatic vascular occlusion, these patients were classified into two groups: IVC clamping and Pringle maneuver (IVCP group, *n* = 15) and Pringle maneuvers alone (Pringle group, *n* = 21) with laparoscopic extracapsular enucleation. Baseline data were similar between the two groups. Patients with hemangiomas that were adjacent to the main portal pedicle, major hepatic veins, or IVC were enrolled in this study. Patient’s clinical records were reviewed, including characteristics of liver hemangiomas, laboratory tests, surgical approaches, intraoperative variables, postoperative complications and mortality, and hospital stay days.

### Basic surgical techniques

**Fig. 1:**
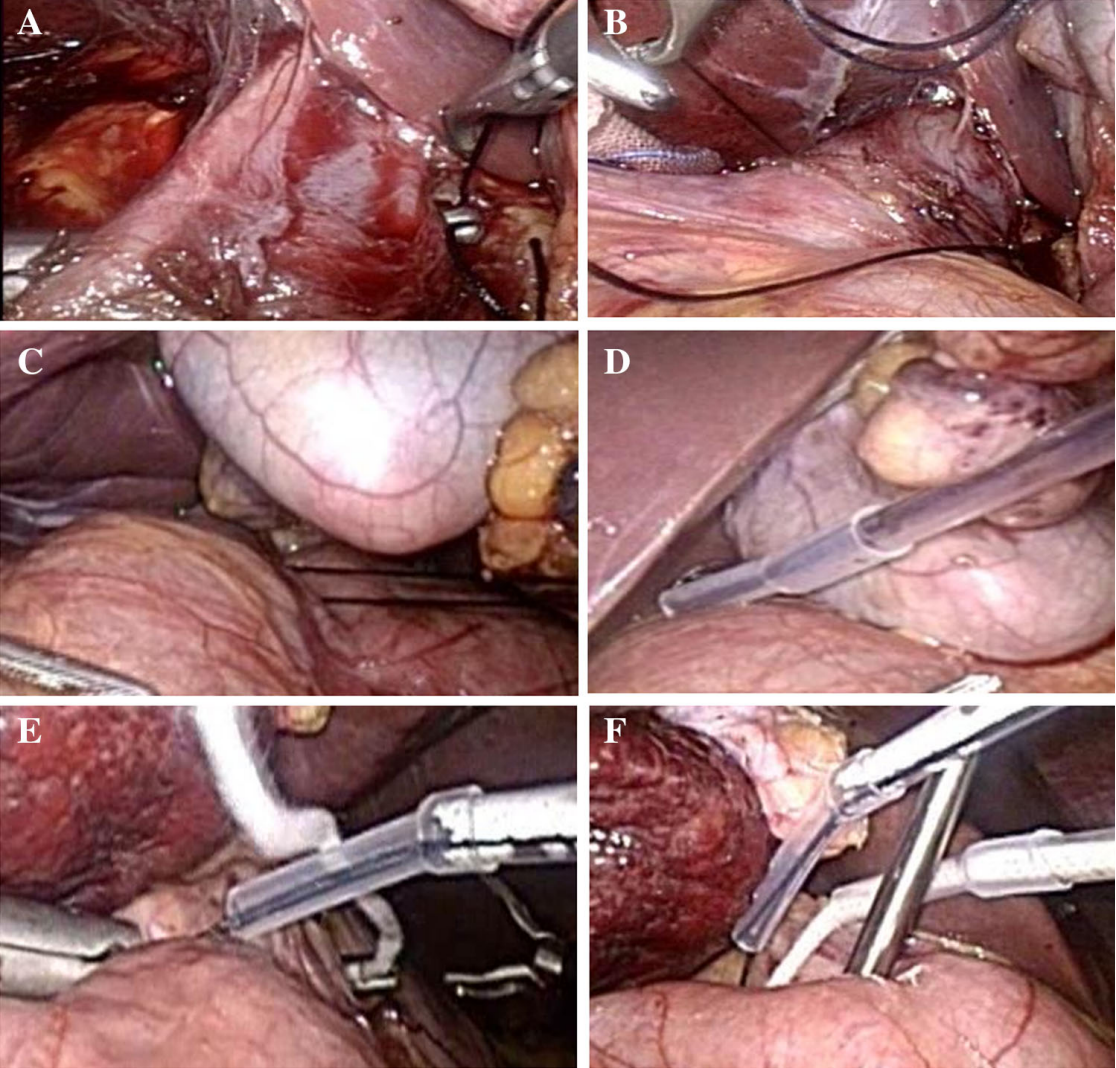
The two hepatic inflow occlusion methods of laparoscopic enucleation for liver hemangiomas. Infrahepatic inferior vena cava clamping: **A** dissected suprarenal IVC above the level of renal vein, and a long vessel forceps was percutaneously inserted into the abdominal cavity and across from the back of infrahepatic inferior vena cava; **B** the forceps nipped a 10/0 surgical string through the infrahepatic inferior vena cava; **C** frap the string smoothly without bending; **D** a 16-Fr hard catheter connected with a 2-cm soft rubber catheter inserted along the string. Pringle maneuver: **E** an umbilical string passed around the hepatoduodenal ligament for inflow occlusion; **F** the progress was the same as infrahepatic inferior vena cava clamping

All of the patients were administered general anesthesia. The patients were positioned in the supine, dorsal elevation. The operating table was tilted 15–30 degrees either the left or the right, depending on the location of the hemangiomas. CO_2_ pneumoperitoneum pressure was established and maintained at 12–14 mmHg. Trocars were distributed around the lesions in a fan-shaped pattern. A five-port method was generally used including a 5-mm trocar, a 10-mm camera port, and a 12-mm trocar which was used for intraoperative ultrasonography and vascular staplers. Before parenchymal resection, an umbilical string was passed through the hepatoduodenal ligament for inflow occlusion (Pringle maneuver). The suprarenal IVC was exposed by dissection of the posterior peritoneum above the level of renal vein, and then a 10/0 surgical string was passed through the infrahepatic IVC. Both strings passed through a 16-Fr hard catheter, and the ends of the hard catheter were connected with a 2-cm soft rubber catheter (Fig. 1). The positional relationship of the main major hepatic veins or portal vein and hemangiomas was identified using intraoperative laparoscopic ultrasound (Aloka, Tokyo, Japan).

Central venous pressure (CVP) was maintained 5 mmHg during liver parenchymal transection. The liver parenchyma was transected with a harmonic scalpel (Ethicon Endo-Surgery, Guaynabo, Puerto Rico). Bipolar electrocoagulation was used for minor bleeding (ERBE). All encountered vessels and bile ducts were transected after clamping with the Hem-o-lok (Weck Surgical Instruments, Teleflex Medical, Durham, NC, USA) or an endoscopic rotation clip (Ethicon Endo-Surgery). Linear laparoscopic staplers (Echelon 60 ENDOPATH Stapler, Ethicon Endo-Surgery, LLC, Cincinnati, OH, USA) were used to transect the major vascular structures along with liver tissues, which can facilitate the parenchymal transection and reduce intraoperative bleeding. The specimen was collected through a 12-mm trocar incision into a plastic bag, and abdominal drains were inserted in place when needed.

### Perioperative data collection

Operative time, clamping time, blood loss, blood transfusion rate, crystalloid fluid infusions, perioperative morbidity, and mortality were evaluated. Serum total bilirubin (TBil), aspartate aminotransferase (AST), alanine aminotransferase (ALT), albumin (Alb), blood urea nitrogen(BUN), and creatinine (Cr) levels were measured on 1, 3, 5, and 7 day(s) after surgery. Postoperative morbidity and mortality were assessed according to the Clavien-Dindo classification.

### Intraoperative hemodynamic parameters

To evaluate the effect of infrahepatic IVC clamping on hemodynamic function, mean arterial pressure (MAP) and heart rate (HR) were measured from the time entered the induction room to the end of surgery.

### Statistical analysis

**Fig. 2:**
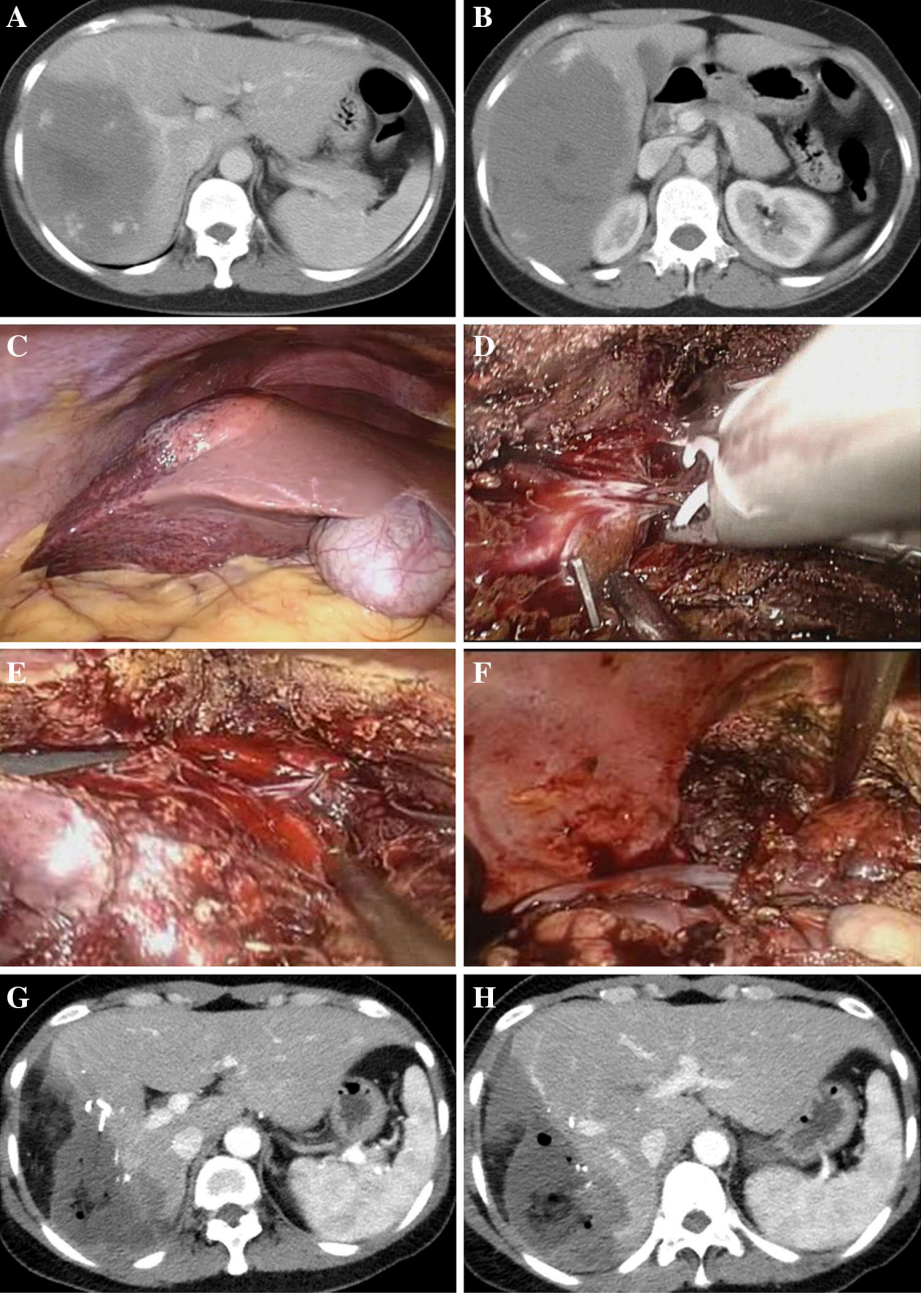
Successfully performed enucleation for giant liver hemangiomas with tumor diameter of 21.7 cm in the right posterior segment and reduced excessive loss of normal liver tissue. **A, B** typical features of hemangioma with contrast-enhanced CT; **C** tumor exterior characteristics with a laparoscopic horizon; **D** branches of middle the hepatic vein ligate with Hem-o-lok; **E** enucleation of hemangiomas along the tumor margin; **F** the cut surface of the remnant liver; **G** postoperative contrast-enhanced CT of tumor enucleation without excessive loss of normal liver tissue; **H** Retained right anterior portal branches without injury

Continuous variables were presented as means and standard deviations. Comparisons were made using the Chi-square test or Fisher’s exact test for categorical data and using the Mann–Whitney *U* test for nonparametric continuous data. All statistical analyses were performed with the SPSS statistical package, version 19.0 (SPSS Inc., Chicago, IL, USA). A value of *p* < 0.05 was considered significant (Fig. 2).

## Results

### Characteristics of patients with giant liver hemangiomas

During January 2012–January 2016, 36 patients with giant liver hemangiomas patients underwent laparoscopic extracapsular enucleation, including 15 with IVC clamping + the Pringle maneuver and 21 with only the Pringle maneuver. Preoperative clinical and laboratory parameters were comparable in both groups of patients (Table 1). There were no significant differences with regard to sex, body mass index, preoperative laboratory test, size, number, location of hemangiomas, and reasons for surgery between the IVCP and the Pringle group (all *p* > 0.05).

**Table 1:** Preoperative clinical features of patients

Variable	Pringle (*n* = 21)	IVCP (*n* = 15)	*p*
Age (years) [mean (SD)]	46.2 ± 10.3	44.2 ± 8.5	0.272
BMI (kg/m2)	23.2 ± 5.7	22.7 ± 5.1	0.357
Gender (M/%)	17 (80.9)	11 (73.3)	0.694
Preoperative laboratory tests			
ALT (U/L)	22.6 ± 8.0	21.4 ± 8.9	0.754
AST (U/L)	22.0 ± 7.4	18.7 ± 6.0	0.241
Albumin (g/L)	34.7 ± 4.0	36.4 ± 4.6	0.358
TBil (µmol/L)	9.9 ± 2.3	9.8 ± 2.6	0.864
BUN (µmol/L)	5.0 ± 1.2	5.2 ± 1.6	0.743
Cr (µmol/L)	60.0 ± 12.8	56.6 ± 7.5	0.415
PT (s)	13.2 ± 0.4	12.1 ± 0.6	0.521
Hb (g/L)	132.7 ± 12.2	131.9 ± 10.8	0.855
PLT (10^9^/L)	203.6 ± 27.3	205.1 ± 29.4	0.902
Fib (g/L)	3.2 ± 0.6	3.4 ± 0.5	0.526
Reasons for surgery treatment			1.00
Symptomatic [*n* (%)]	18 (85.7)	14 (93.3)	
Rupture	0	0	
Increasing growth [*n* (%)]	2 (9.5)	1 (6.7)	
K–M syndrome [*n* (%)]	1 (4.8)	0	

Values are mean (SD)

Comparisons were made using the Chi-square test or Fisher’s exact test or Mann–Whitney *U* test as appropriate

*ALT* alanine aminotransferase, *AST* aspartate aminotransferase, *Alb* albumin, *TBil* total bilirubin, *BUN* blood urea nitrogen, *Cr* creatinine, *PT* prothrombin time, *Hb* hemoglobin, *PLT* platelet

### Intraoperative outcomes

There were no significant differences between two the groups in the operative time (259.5 vs 282.2 min) and the Pringle clamping time (31.3 vs 26.4 min). The median IVC clamping time was 14.2 min (range 5–20 min). Total median blood loss was significantly lower in IVCP group than in the Pringle group (315.3 vs 586.7 mL, *p* < 0.001). Five patients received an intraoperative transfusion in the IVCP group, and the transfusion rate was higher than that in the Pringle group. Compared with the Pringle group which had 9.5% conversion rate, no patients encountered conversion in IVCP group. The intraoperative data are shown in Table 2.

**Table 2:** Intraoperative data of hepatectomy for patients

Variable	Pringle (*n* = 21)	IVCP (*n* = 15)	*p*
Tumors number [*n* (%)]			1.00
Single	15 (71.4)	11 (78.6)	
Multiple	6 (28.6)	4 (21.4)	
Tumor size (cm)	12.9 ± 3.4	14.8 ± 6.2	0.444
Tumor location [*n* (%)]			0.506
Right lobe	17 (81.0)	12 (80.0)	
Left lobe	1 (4.7)	2 (13.3)	
Bilateral	3 (14.3)	1 (6.7)	
Operation time (min)	259.5 ± 36.0	282.2 ± 53.0	0.376
Pringle time (min)	31.3 ± 6.2	26.4 ± 5.7	0.296
IVC clamping time	0	14.2 ± 1.6	
Total blood loss (mL)	586.7 ± 217.0	315.3 ± 86.3	**<0.001**
Enucleation-related blood loss (mL)	473.9 ± 201.5	203.3 ± 76.6	**<0.001**
Crystalloid fluid infusions (mL)	1733.3 ± 359.4	1683.3 ± 250.0	0.237
Transfusion rate [*n* (%)]	5 (23.8)	1 (6.7)	0.367
Conversion rate [*n* (%)]	2 (9.5)	0	0.500

Bold values indicate statistically significant

Comparisons were made using Chi-square test or Fisher’s exact test or Mann–Whitney *U* test as appropriate

### Intraoperative hemodynamic parameters

The incidence of hemodynamic instability occurred more frequently in the IVC clamping group than that in the Pringle group. The two groups showed a decrease in MAP and an increase in HR. In the Pringle group, variation of MAP after and before the Pringle clamping did not show statistically significant difference (91.1 vs 94.3 mmHg, *p* = 0.093), while MAP significantly decreased after hepatic IVC clamping as compared to pre-clamping level (70.9 vs 93.5 mmHg, *p* < 0.001). In both groups, HR of patients significantly increased after vascular clamping (*p* < 0.001). After unclamping the infrahepatic IVC, MAP and HR returned to normal levels. Hemodynamic changes are presented in Table 3.

**Table 3:** Comparison of intraoperative hemodynamic changes with vascular clamping

Hemodynamic changes (mmHg)	MAP			HP		
	BC	AC	*p*	BC	AC	*p*
Pringle	94.3 ± 6.4	91.1 ± 5.3	0.093	71.2 ± 7.7	80.5 ± 6.1	**0.001**
IVCP	93.5 ± 5.7	70.9 ± 6.1	**<0.001**	73.3 ± 13.0	92.6 ± 11.0	**<0.001**

Bold values indicate statistically significant

Comparisons were made using Mann–Whitney *U* test

*MAP* mean arterial pressure, *BP* blood pressure, *BC* before clamping, *AC* after clamping, *HP* heart rate

### Postoperative outcome parameters

**Fig. 3:**
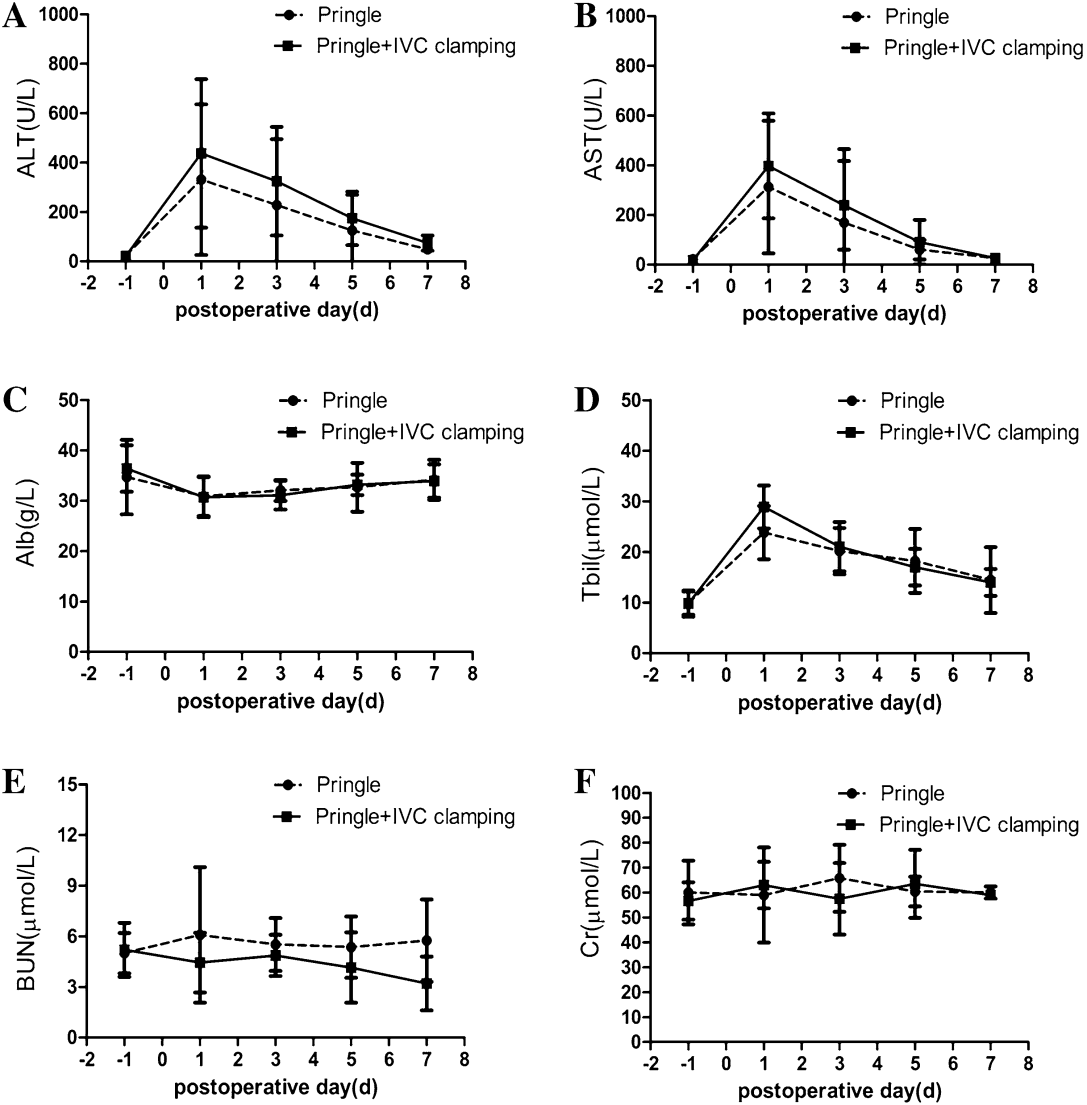
The postoperative tests on 1, 3, 5, and 7 day(s) in the Pringle group and IVCP groups. **A** *ALT* alanine aminotransferase, **B** *AST* aspartate aminotransferase, **C** *Alb* albumin, **D** *TBil* total bilirubin, **E** *BUN* blood urea nitrogen, **F** *Cr* creatinine

In our patients, there were no significant differences between the two groups in the rates of postoperative complications (Table 4). The major complications of the two groups were pleural effusion and peritoneal effusions. Of the 36 patients, 16 patients had pleural effusion, 14 with peritoneal effusions, which could be treated conservatively. There was no IVC clamping-related morbidity and no mortality in both groups, and the two groups required almost the same length of hospitalization. Except for portal triad clamping, liver function could also be impaired by the IVC clamping. The serial changes in the serum ALT, AST, ALB, and TBil level did not show significant difference between two groups. Because the IVC was fully blocked, there was a risk of renal impairment due to venous blood congestion. However, there were no significant differences in intraoperative urine volume, postoperative BUN, and creatinine values between the two groups (Fig. 3).

**Table 4:** Comparison of postoperative complication and outcomes

Variable	Pringle (*n* = 21)	IVCP (*n* = 15)	*p*
*Complications*			
Grade 1			
Wound infection	0	0	
Grade 2			
Pleural effusions [*n* (%)]	10 (46.7)	6 (40.0)	0.741
Ascites [*n* (%)]	9 (42.9)	5 (33.3)	0.732
Grade 3			
Pulmonary problems	1	0	
PTE	0	0	
VT	0	0	
Biloma/bile leak	0	0	
Intra-abdominal bleeding	0	0	
Grade 4			
Organ dysfunction	0	0	
Grade 5			
Mortality	0	0	
Hospital stay (day)	9.6 ± 2.1	8.8 ± 3.5	0.807

Comparisons were made using Chi-square test or Fisher’s exact test or Mann–Whitney *U* test as appropriate

*PTE* pulmonary thromboembolism, *VT* venous thrombosis

## Discussion

Surgical intervention, principally including liver anatomical resection and tumor enucleation, is the definitively curative treatment for liver hemangiomas. Recent advances in surgical techniques and established perioperative management have made liver resection or enucleation safe in most specialized units. When surgery is indicated, extracapsular enucleation is advocated by most surgeons. This surgical option can retain the maximum amount of normal liver parenchyma, minimize intraoperative blood loss, and decrease the incidence of postoperative biliary fistula in open surgery. However, with some liver hemangiomas, the distress caused by open surgery may be far worse than the tumor-related discomfort. Therefore, we prefer the laparoscopic approach for the treatment of hemangiomas.

With the progress of minimally invasive techniques, laparoscopic surgery for hepatic procedures has been rapidly developing. More complicated laparoscopic liver resections are now possible, including right hepatectomies, left hepatectomies, and even extended right and left hepatectomies [16, 17]. Compared with open surgical resection, laparoscopic resection offers many advantages, such as a shorter hospital stay, improved cosmetic results, and potential improvements to immunological outcomes [18, 19]. In order to reduce bleeding, in previous studies, laparoscopic anatomical liver resection was used for the removal of hepatic hemangiomas, but the procedure tends to result in excessive loss of liver tissues or even gallbladder [20]. In our study, 36 cases of liver hemangiomas were adjacent to the main portal pedicle, major hepatic veins, or gallbladder that previously recommended anatomical liver resection in laparoscopic hepatectomy. Nevertheless, we performed enucleation for all of the cases of liver hemangiomas. Enucleation has retained as much as possible of liver tissue, while giant hepatic hemangioma often close to the hepatic vein or portal vein leads to excessive bleeding especially for laparoscopic hepatic hemangioma enucleation and even results in the conversion to open surgery. Therefore, the hepatic vascular occlusion for bleeding control is critical to hepatectomy [21]. The Pringle maneuver could minimize hepatic arterial and portal blood flow to liver during transection of hepatic parenchyma; actually, a major cause of blood loss during liver resection is often attributed to the injury of hepatic vein [22]. Compared with bleeding from the portal vein and hepatic artery, hemorrhage from the injured hepatic vein is much more difficult to control throughout the whole laparoscopic hepatectomy procedure.

To decrease bleeding from the hepatic vein, low CVP which is controlled by anesthesiological methods has gained wide acceptance among hepatobiliary surgeons, based on the results of various randomized studies in the field of liver surgery [23, 24]. However, this finding has been contradicted by some other studies in which CVP monitoring did not appear to reduce blood loss in elective liver resection and globally, the intraoperative clinical value of CVP was questioned [25]. Rahbari et al. and our studies showed that infrahepatic clamping of the IVC significantly reduced intraoperative blood loss compared with anesthesiological methods of low CVP and that it did not reveal a significant association between the CVP and intraoperative blood loss irrespective of the applied method of reduction of CVP [15, 26]. In addition, infrahepatic IVC clamping may considerably reduce the adverse effects of reduction of CVP by anesthesiological interventions and the risk of intraoperative bleeding primarily from the hepatic veins [27, 28]. In this study, using IVC clamping to control blood loss, we were in a position to precisely identify the major vascular architecture around the hemangioma and selectively clip or ligate it, thus limiting blood loss during liver transection [14]. With fewer blood vessels traversing the capsule, the tumor can be enucleated without much blood loss by cutting open this layer between the capsule of the hemangioma and the liver. In our experience, the dissection of infrahepatic IVC is not so difficult and can be completed within a relatively short time (7–21 min). In the current study, we confirmed the advantages of infrahepatic IVC clamping to reduce blood loss under laparoscopic hepatectomies. Patients in the IVCP group experienced less blood loss and fewer blood transfusion requirements than those in the Pringle group. The total amount of blood loss in the IVCP group was significantly less than that in the Pringle group, and no patient in IVCP group had more than 1000 mL of blood loss during the operation.

Safety of portal triad clamping during liver resection has not been a matter of debate. IVC clamping technique with the Pringle maneuver has been thought to increase hemodynamic instability and postoperatively elevate the serum level of alanine aminotransferase, which can be deleterious in major liver resection in a diseased liver. In most cases, clamping IVC technique was well tolerated. The IVC was encircled at a site proximal to the bilateral renal veins; there was the potential risk of renal function impairment due to venous blood congestion [29]. However, there were no significant differences in intraoperative urine volume, postoperative BUN, and creatinine values between the groups. Furthermore, all of the liver function profile, such as AST, ALT, and TBil, did not fluctuate substantially and returned to normal ranges within a short period of time after operation. The risk of laparoscopic hepatectomy is CO_2_ gas embolism (gas embolism), which is associated with pneumoperitoneum gas influx into the IVC or hepatic vein during liver parenchymal transection [30, 31]. Our experience showed that the enucleation of hepatic hemangioma mostly did not expose the inferior vena cava and short hepatic veins and their injury was comparatively less likely. The hepatic vein perhaps may encounter a small amount of carbon dioxide influx into the blood, but generally, this will not lead to an embolism. As the operation is carried out in a blood loss surgical field, we can easily clamp or control the bleeding from the hepatic vein with infrahepatic IVC clamping. To date, we have performed 36 patients with hepatic hemangioma surgery under laparoscopic resection with IVC clamping and Pringle maneuver. So far, we have performed roughly 80 such operations without any serious complications. In future, we will conduct further studies in this regard.

Our study had limitations. Intraoperative blood loss according to the type of clamping procedures was not assessed, because of the retrospective nature of this study. Therefore, it was unable to demonstrate any superiority of one clamping procedure upon another.

## Conclusion

Infrahepatic clamping of the IVC and the Pringle maneuver significantly reduces intraoperative blood loss compared with the Pringle maneuver alone. IVC clamping and the Pringle maneuver may serve as a safe, effective, and feasible technique for enucleation of hepatic hemangioma.

## Abbreviations

IVC: Inferior vena cava
IVCP: IVC clamping and the Pringle maneuvers
CVP: Central venous pressure
SD: Standard deviation
ALT: Alanine aminotransferase
AST: Aspartate aminotransferase
Alb: Albumin
TB: Total bilirubin
BUN: Blood urea nitrogen
Cr: Creatinine
PT: Prothrombin time
Hb: Hemoglobin
MAP: Mean arterial pressure
HP: Heart rate
